# The Validity of Impressions as a Media Dose Metric in a Tobacco Public Education Campaign Evaluation: Observational Study

**DOI:** 10.2196/55311

**Published:** 2024-11-05

**Authors:** Kevin Davis, Laurel Curry, Brian Bradfield, David A Stupplebeen, Rebecca J Williams, Sandra Soria, Julie Lautsch

**Affiliations:** 1 RTI International Research Triangle Park, NC United States; 2 RTI International Washington, DC United States; 3 Office of Public Health Studies Thompson School of Social Work and Public Health University of Hawaiʻi at Mānoa Honolulu, HI United States; 4 California Tobacco Prevention Program California Department of Public Health Sacramento, CA United States

**Keywords:** communication, public education, tobacco, media, public health

## Abstract

**Background:**

Evaluation research increasingly needs alternatives to target or gross rating points to comprehensively measure total exposure to modern multichannel public education campaigns that use multiple channels, including TV, radio, digital video, and paid social media, among others. Ratings data typically only capture delivery of broadcast media (TV and radio) and excludes other channels. Studies are needed to validate objective cross-channel metrics such as impressions against self-reported exposure to campaign messages.

**Objective:**

This study aimed to examine whether higher a volume of total media campaign impressions is predictive of individual-level self-reported campaign exposure in California.

**Methods:**

We analyzed over 3 years of advertisement impressions from the California Tobacco Prevention Program’s statewide tobacco education campaigns from August 2019 through December 2022. Impressions data varied across designated market areas (DMAs) and across time. These data were merged to individual respondents from 45 waves of panel survey data of Californians aged 18-55 years (N=151,649). Impressions were merged to respondents based on respondents’ DMAs and time of survey completion. We used logistic regression to estimate the odds of respondents’ campaign recall as a function of cumulative and past 3-month impressions delivered to each respondent’s DMA.

**Results:**

Cumulative impressions were positively and significantly associated with recall of each of the Flavors Hook Kids (odds ratio [OR] 1.15, *P*<.001), Dark Balloons and Apartment (OR 1.20, *P*<.001), We Are Not Profit (OR 1.36, *P*<.001), Tell Your Story (E-cigarette, or Vaping, product use Associated Lung Injury; OR 1.06, *P*<.05), and Thrown Away and Little Big Lies (OR 1.05, *P*<.01) campaigns. Impressions delivered in the past 3 months were associated with recall of the Flavors Hook Kids (OR 1.13, *P*<.001), Dark Balloons and Apartment (OR 1.08, *P*<.001), We Are Not Profit (OR 1.14, *P*<.001), and Thrown Away and Little Big Lies (OR 1.04, *P*<.001) campaigns. Past 3-month impressions were not significantly associated with Tell Your Story campaign recall. Overall, magnitudes of these associations were greater for cumulative impressions. We visualize recall based on postestimation predicted values from our multivariate logistic regression models.

**Conclusions:**

Variation in cumulative impressions for California Tobacco Prevention Program’s long-term multichannel tobacco education campaign is predictive of increased self-reported campaign recall, suggesting that impressions may be a valid proxy for potential campaign exposure. The use of impressions for purposes of evaluating public education campaigns may help address current methodological limitations arising from the fragmented nature of modern multichannel media campaigns.

## Introduction

Evaluations of mass media tobacco education campaigns have often used variation (over time and across markets) in campaign target ratings points (TRPs) or gross ratings points (GRPs) to estimate dose-response relationships between campaign advertisement buys and outcomes of interest (eg, Davis et al [1]; Farrelly et al [2]; Wakefield et al [3]). Ratings points are based on Nielsen ratings for programs during which TV or radio advertisements are shown, providing a metric of population-level reach and frequency of exposure to a given ad for specific audiences [4]. These metrics vary by designated market areas (DMAs) and over time. Because these metrics assume that individuals within a market’s population have the same opportunity to view a campaign’s advertisements, these metrics are only applicable for broadcast media channels [5].

An advantage of using GRPs or TRPs for media evaluation is that variation in ratings data is exogenous to study respondents and therefore not subject to potential selective attention biases that can arise from using self-reported measures of ad exposure. As such, this approach enables stronger conclusions about causality of campaign effects. However, a major limitation of this approach is that ratings data typically only capture delivery of broadcast media (TV and radio) and exclude other media channels. Therefore, this method is only applicable to evaluations of media campaigns that predominantly rely on broadcast channels in their media buys. In addition, the delivery of media content has shifted dramatically over the past decade toward a more fragmented system that simultaneously uses multiple nonbroadcast channels, including digital video, digital displays, and streaming audio. These channels are often used on their own or in combination with traditional broadcast media.

These changes to the modes and channels of media distribution complicate the evaluation of modern public health media campaigns. Traditional evaluation methods that include the use of GRPs may not adequately capture the breadth of a campaign’s media delivery and related variation in that delivery. A recent review by Durkin et al [6] cataloged many of these challenges in the tobacco prevention media landscape, calling for an exploration of alternative methods and data to measure total campaign exposure across the spectrum of media channels that are used. These authors noted that studies to “validate self-reported exposure to digital campaign messages against objective digital metrics such as impressions, advertisements served, and completed video views” are needed [5-8].

Previous research has examined the relationships between digital tobacco education campaigns and audience response, including recall of advertisements and information seeking. For example, Kim et al [7] found that consumers who were verified as being exposed to a set of digital tobacco education advertisements were significantly more likely than unexposed consumers to visit websites that were promoted by the campaign. Other studies have used intentional geography-based manipulation of ad buys to show that markets receiving additional doses of digital video had higher overall campaign awareness compared with markets that received a conventional dose of standard TV and digital media [8]. Although these studies demonstrate correlation between nonbroadcast media delivery and overall campaign reach, they do not provide a basis for establishing alternatives to TRPs or ratings-based metrics for measuring overall delivery doses of a multichannel campaign. Validating the predictive relationships between an alternative comprehensive metric for ad delivery, such as impressions, and self-reported exposure to campaign advertisements requires more robust datasets with frequent collection of self-reported exposure merged with high variation in ad delivery metrics.

In this study, we use data from the California Tobacco Prevention Program’s (CTPP’s) tobacco education media campaign to examine the relationship between self-reported exposure to campaign messages and total campaign impressions across all channels. CTPP’s statewide campaign is a dynamic multimessage campaign that targets numerous audiences using TV, radio, digital video, digital audio and display, and other nonbroadcast channels. In addition, CTPP’s campaign ad buys are predominantly purchased based on guaranteed impressions units, not promised TRPs. As part of its broader campaign evaluation, CTPP tracks self-reported exposure to campaign messages and other outcomes using a large monthly survey of adults in California. Our study merges this robust survey data with detailed information on campaign impressions over time and across media markets to examine whether higher volume of total campaign impressions is predictive of individual-level self-reported campaign exposure in California. We discuss the implications and future directions of this research for using impressions as a valid exogenous proxy for total exposure to multichannel campaigns.

## Methods

### Survey Data

Survey data came from a monthly web-based tracking survey recruited from the Dynata consumer panel, a large commercially available panel of US adults. The data were collected in 45 waves from August 2019 through December 2022 and included respondents from California between 18 and 55 years of age (N=151,649). Dynata is a nonprobability convenience panel that uses a range of recruitment approaches, including email and marketing as well as targeted websites to yield a broad sample of US consumers. Dynata panelists are remunerated through an e-rewards system that provides redeemable points for merchandise for each completed survey. Each web survey was offered in English and Spanish to accommodate Spanish-speaking respondents who speak limited or no English or preferred to take the survey in Spanish. Sample sizes varied by wave, ranging from 2954-3576 per wave. Respondents were allowed to participate in more than 1 wave (34.9% of observations (n=52,971) were from repeat respondents). The survey data were weighted and calibrated to reflect distributions (within Census region) of sex, age, race and ethnicity, education, smoking status (smoker vs nonsmoker), and daily internet use.

### Measures

#### Recall of California Tobacco Prevention Program Campaigns

Recall of each of 5 CTPP campaigns that aired over the course of survey data collection from August 2019 to December 2022 were the primary outcome variables for this study: Flavors Hook Kids, We Are Not Profit, Dark Balloons and Apartment, Tell Your Story, and Thrown Away and Little Big Lies. The Flavors Hook Kids and We Are Not Profit campaigns highlighted the effects of flavored tobacco products on youth and Black populations and deceptive industry marketing tactics, among other themes. The Dark Balloons and Apartment campaigns focused on the dangers and ubiquity of secondhand smoke, particularly in outdoor and multiunit housing settings. CTPP’s Tell Your Story campaign was focused on providing young adults with vaping cessation services in response to the E-cigarette or Vaping-Associated Lung Injury outbreak, while the Thrown Away and Little Big Lies campaigns highlighted the negative effects of discarded tobacco products, including the proliferation of microplastics in the environment. More information about CTPP’s media campaigns can be found on the program’s website [9].

##### Unaided Campaign Recall

We assessed unprompted general recall of the CTPP campaign to gauge overall awareness of the campaign in California. Respondents were asked “Have you recently seen or heard any California Department of Public Health antitobacco ads on TV, online, on radio, in magazines or on outdoor signs?” with response options of “yes” and “no.” Respondents who answered “yes” were considered to have unaided recall of the overall California Department of Public Health antitobacco campaign.

##### Prompted Ad Recall

Specific ad recall was measured using an aided ad recognition protocol where after viewing videos of each campaign ad, respondents were asked “Have you seen this ad on TV or online?” Respondents could answer “yes” or “no” to each ad-specific recall question. A similar protocol was used to construct recall of radio advertisements. After listening to each radio advertisement (or reading the script if the ad playback function did not work properly), respondents were asked “Have you heard this ad on the radio?” with response options of “yes” and “no.” Respondents were also asked to recall whether they had seen any outdoor or online static display-style advertisements, through the following prompt: “Next, we would like to show you an image of a few ads that may have appeared on outdoor signs in California such as on buses, billboards, gas stations, or public places, or on websites and other places online.” After viewing images of these advertisements, respondents selected “yes” or “no” to indicate having seen the advertisements previously or not. We then created campaign-specific awareness variables indicating awareness of at least one ad across all ad types (video, radio, or static). These campaign-specific ad recall variables served as the dependent variables in each of our analytic models.

### Exogenous Campaign Exposure Measured by Impressions

We used weekly impressions data provided by the campaign’s media buyer to measure potential exposure to CTPP’s paid media. Impressions are defined as the number of times a piece of media displays on a user’s screen (or plays on an audio channel). These data were provided at the DMA level for the 14 DMAs in California and were calculated separately for each campaign aired by CTPP across multiple target audiences of adults in California. Impressions were included for each media channel used by the campaign (broadcast, digital, and display) and combined to provide a compilation of total potential exposure across all channels. We measured total cumulative impressions and past 3-month impressions for each of CTPP’s campaigns on air during this time period: Flavors Hook Kids, Dark Balloons and Apartment, Tell Your Story, Thrown Away and Little Big Lies, and We Are Not Profit campaigns. Impression values were linked to individual survey respondents based on survey timing and respondent location in DMAs. Each survey participant was assigned the full week of impressions data for the week they took the survey, no matter which day of the week they participated. Cumulative impressions were totaled from campaign launch through the date each participant took the survey for the DMA in which they reported residing. Past 3-month impressions were similarly totaled, but from the 3 months before the respondent’s survey date.

Mean values of cumulative and past 3-month impressions by campaign are summarized in Table 1. While these values reflect the market-level mean impressions, respondents in the sample can experience a range of impression levels (from low to high) based on the market where they reside.

**Table 1:** Mean Market-level California Tobacco Prevention Program’s cumulative campaign impressions, August 2019 to December 2022.

Campaign	Cumulative impressions, mean (SD)^a^	Past 3-month impressions, mean (SD)
All campaigns	328,789,592 (586,912,213)	42,758,557 (90,205,224)
Flavors Hook Kids	152,422,867 (238,477,527)	17,052,572 (40,158,606)
Dark Balloons and Apartment	112,884,348 (208,276,945)	9,870,736 (46,101,224)
We Are Not Profit	40,224,813 (99,809,204)	9,413,460 (31,246,961)
Tell Your Story	13,277,377 (39,494,383)	2,733,164 (12,124,264)
Thrown Away and Little Big Lies	9,980,187 (38,762,599)	3,688,625 (20,496,183)

^a^There were 176 weeks and 14 unique DMAs (n=2464 weeks x DMA observations) represented in the analytic data.

### Control Variables

Our analysis controlled for several factors that may influence recall of campaign advertisements. We created control variables for respondent demographics including age (18-55 years); race and ethnicity (White non-Hispanic, Black non-Hispanic, Asian non-Hispanic, other non-Hispanic, and Hispanic); gender (male, female, transgender, or other or unsure); sexual orientation (straight or do not know and LGBQ [lesbian, gay, bisexual, and queer/questioning]); education (less than high school, high school degree, some college, associate degree, bachelor’s degree or higher); and income (low income—less than US $45,000, middle to high income—US $45,000 or more). We also controlled for any past 30-day use of tobacco products (including e-cigarettes) or marijuana. Finally, we controlled for background secular trends in campaign recall by measuring total quarters elapsed since the beginning of the study (August 2019).

### Statistical Analysis

We began by summarizing average campaign recall for each of the CTPP campaigns described earlier. Campaign recall was estimated separately for each year in our data (2019-2022) and for all years combined. We then used logistic regression to estimate the odds of campaign-specific recall as a function of cumulative and past 3-month impressions delivered to each respondent’s DMA. Impressions were scaled to produce odds ratios that reflected the increase in campaign recall for delivery of the mean past 3-month and cumulative impressions levels. This scaling provides a realistic interpretation of the relationship between impressions and campaign recall based on mean levels of impressions delivered for each campaign in the real world. In addition, because we did not expect increasing levels of paid impressions to perpetually generate linear increases in recall, we used a nonlinear functional form for impressions (square root) to capture asymptotic diminishing effects over the range of observed impressions. Each model included control variables for the respondent characteristics described earlier, as well as a linear time trend for quarters since the first survey wave of the study in August 2019.

To examine whether channel-specific impressions were more or less strongly predictive of campaign recall, we conducted the same analysis using 3 separate models: one with just video impressions, another with just audio impressions, and another with just display impressions. We then compared the odds ratios (ORs) across those 3 model types to determine whether different impression types were more strongly predictive of overall recall than others.

To further illustrate the estimated relationship between campaign impressions and recall, we calculated postestimation predicted probabilities of overall prompted campaign recall across observed values of past 3-month cumulative impressions for the combined CTPP campaigns. We chose to use past 3-month impressions as the basis for these predictions as this is a more common time frame for real-world ad buys. These predictions were generated from the multivariate logistic regression model results to provide interpretable ranges of campaign recall across the observed range of cumulative impressions with all covariates in our statistical model held constant. Because a small number of respondents in certain markets receive very high impressions levels, these predictions exclude the highest 5% of impression values. This helps ensure that our graphical representation of the dose-response relationship between impressions and ad recall reflects the experience of most respondents and is not skewed by outlier impressions values. All analyses were conducted using Stata (version 17.0, Computing Resource Center).

### Ethical Considerations

RTI International’s Institutional Review Board reviewed this research protocol and determined that it did not meet the definition of research with human subjects because it was for program evaluation purposes. Nevertheless, all study participants gave informed consent for panel participation in addition to assurances that all study participation is voluntary, and any question could be skipped if desired.

## Results

### Sample Characteristics

The combined unweighted sample included 151,649 observations on adults aged 18 to 55 years in California collected from August 2019 to December 2022. The mean unweighted age of the sample was 38.9 years and consisted of individuals across the following demographics: 69,627 White non-Hispanic (45.9%), 14,904 Black non-Hispanic (9.8%), 23,812 Asian non-Hispanic (15.7%), 8436 other non-Hispanic races (5.6%), and 34,858 Hispanic (23.0%) adults. The unweighted and weighted sample distributions of gender, sexual orientation, education, income, and tobacco and other product use are summarized in Table 2.

**Table 2:** Unweighted sample demographics and other characteristics.

Respondent characteristic				Unweighted percentage		Weighted percentage		Participants, n
**Age (years), mean**				38.92		36.27		—^a^
		18-29	18.7		31		28,360	
		30-55	81.3		69		123,289	
**Race and Ethnicity**								
	White non-Hispanic			45.9		34.7		69,627
	Black non-Hispanic			9.8		6.1		14,904
	Asian non-Hispanic			15.7		17.5		23,812
	Other non-Hispanic			5.6		1.4		8436
	Hispanic			23.0		40.2		34,858
**Gender**								
	Male			40.7		49.1		61,577
	Female			57.9		48.8		87,595
	Transgender			0.7		1.3		1091
	Other			0.5		0.5		717
	Unsure			0.2		0.3		267
**Sexual orientation**								
	Straight or do not know			87.7		86.7		130,404
	LGBQ^b^			12.3		13.3		18,252
**Education**								
	Less than high school			2.9		15.4		4446
	High school degree			14.8		21.6		22,455
	Some college			21.6		20.7		32,750
	Associate degree			11.9		11.3		18,088
	Bachelor’s degree or higher			48.7		31.1		73,902
**Income**								
	Low income (less than US $45,000)			33.7		45.4		51,042
	Middle to high income (US $45,000 or more)			66.3		54.6		100,471
**Tobacco and other product use**								
	Smoked cigarettes in past 30 days			30.3		9.4		45,946
	Used electronic vape products in past 30 days			25.4		13.6		38,520
	Used marijuana in past 30 days			32.5		22.3		49,321
	Used other tobacco products in past 30 days			23.2		14.5		35,200

^a^—: not applicable.

^b^LGBQ: lesbian, gay, bisexual, and queer/questioning.

### Campaign Recall

Table 3 summarizes descriptive statistics on campaign recall by year from 2019 to 2022. Across all years in our analysis, 68.3% (53,137) of respondents indicated awareness of any ad in the CTPP Flavors Hook Kids campaign. During this time frame, recall of the We Are Not Profit campaign was 59.1% (16,920) while recall of the Dark Balloons and Apartment, Tell Your Story, and Thrown Away and Little Big Lies campaigns were 54.8% (27,748), 53.2% (16,886), and 50.8% (15,450), respectively. Overall, 65.6% (87,814) of adults aged 18 to 55 years old in California reported awareness of any CTPP campaign ad across the combined time period from 2019 to 2022. Unprompted general recall of antitobacco advertisements in California across all years was 51.5% (78,090).

**Table 3:** Recall of California Tobacco Prevention Program campaigns among all adults aged 18-55 years old in California, August 2019 to December 2022.

Campaign			2019 (n=19,004)		2020 (n=45,509)		2021 (n=39,584)		2022 (n=47,552)		All years (n=151,649)
**Prompted Ad Recall**											
	All campaigns	60.9%		67.7%		68.5%		62.7%		65.6%	
	Flavors Hook Kids	65.8%		65.4%		74.1%		67.0%		68.3%	
	We Are Not Profit	—^a^		47.8%		62.4%		—^a^		59.1%	
	Dark Balloons and Apartment	51.4%		59.2%		49.9%		—^a^		54.8%	
	Tell Your Story	—^a^		—^a^		47.5%		58.8%		53.2%	
	Thrown Away and Little Big Lies	—^a^		49.3%		—^a^		51.5%		50.8%	
	Unaided campaign recall	56.5%		49.4%		49.7%		53.1%		51.5%	

^a^—: not applicable.

### Relationship Between Campaign Impressions and Recall

Cumulative and past 3-month impressions were widely associated with prompted campaign recall (Table 4). Cumulative impressions were positively and significantly associated with recall of each of the Flavors Hook Kids (OR 1.15, *P*<.001), Dark Balloons and Apartment (OR 1.20, *P*<.001), We Are Not Profit (OR 1.36, *P*<.001), Tell Your Story (OR 1.06, *P*<.05), and Thrown Away and Little Big Lies (OR 1.05, *P*<.01) campaigns. Impressions delivered in the past 3 months were associated with recall of the Flavors Hook Kids (OR 1.13, *P*<.001), Dark Balloons and Apartment (OR 1.08, *P*<.001), We Are Not Profit (OR 1.14, *P*<.001), and Thrown Away and Little Big Lies (OR 1.04, *P*<.001) campaigns. Past 3-month impressions were not significantly associated with Tell Your Story campaign recall. Overall, magnitudes of these associations were greater for cumulative impressions.

Similar associations between impressions and unaided campaign recall were found. Past 3-month impressions (OR 1.07, *P*<.001) and cumulative impressions (OR 1.05, *P*<.01) were both significantly associated with unaided campaign recall.

**Table 4:** Odds ratios and 95% CIs from logistic regressions of associations between campaign impressions and campaign recall among all adults in California aged 18-55 years, August 2019-December 2022.

Prompted ad recall	Cumulative impressions, OR (95% CI)	Past 3-month impressions, OR (95% CI)
All campaigns	1.16^a^ (1.11-1.20)	1.20^a^ (1.17-1.23)
Flavors Hook Kids	1.15^a^ (1.09-1.22)	1.13^a^ (1.10-1.17)
Dark Balloons and Apartment	1.20^a^ (1.13-1.27)	1.08^a^ (1.06-1.11)
We Are Not Profit	1.36^a^ (1.27-1.46)	1.14^a^ (1.09-1.20)
Tell Your Story	1.06^b^ (1.01-1.11)	1.01 (0.98-1.04)
Thrown Away and Little Big Lies	1.05^c^ (1.01-1.09)	1.04^a^ (1.02-1.07)
Unaided campaign recall	1.04^c^ (1.01-1.08)	1.08^a^ (1.05-1.10)

^a^*P* (overall): <.001.

^b^*P* (overall): <.05.

^c^*P* (overall): <.01.

We divided the impressions variables used in the models by the overall sample mean to produce odds ratios that give the percent change in recall for a unit change in impressions where the unit change is the sample mean of the impressions. To illustrate, for cumulative impressions, going from no campaign (ie, 0 impressions) to the average campaign delivery (328 million impressions) generates a 16% increase in odds of ad recall. This scaling provides a realistic unit of change for impressions that reflects the size of a real-world ad buy for the CTPP campaign. Results from our sensitivity analysis showed similar relationships between the channel-specific impressions (video, audio, and display) and campaign awareness measured through both prompted and unaided recall (results not shown). Given that CTPP used a relatively balanced mixture of these media channels concurrently, combined impression should best reflect the overall relationship between campaign advertisements buys and reach among target audiences in California.

Multimedia Appendix 1 summarizes estimated campaign recall rates based on postestimation predicted values from our multivariate logistic regression model for overall campaign recall. Predicted recall for all campaigns combined ranged from 63.7% at 100 million past 3-month cumulative impressions to 77.0% at 800 million past 3-month cumulative impressions.

## Discussion

### Principal Findings

Results from this study show that variation in cumulative impressions for CTPP’s long-term multichannel tobacco education campaign is predictive of increased self-reported campaign recall, suggesting that impressions may be a valid proxy for potential campaign exposure. Because impressions are generally calculated and known for all media channels, the use of impressions for purposes of evaluating public education campaigns may help address current methodological limitations arising from the fragmented nature of modern multichannel media campaigns [6]. That is, impressions can provide a single metric that captures whole-campaign exposure regardless of a campaign’s media channel mixture. The use of impressions may be particularly helpful in the evaluation of campaigns that dedicate substantial portions of ad buys to nonbroadcast channels, including digital video, display, and out-of-home advertising.

Our study also provides one of the first real-world examples of how impressions data can be applied in the evaluation of a major multichannel public health awareness campaign. In our application of these data, there is a high degree of variation in impressions over a long period of time. Impressions variation, in this context, arises from differences in ad buy sizes across media markets and from time-specific accumulation of impressions. This variation in impressions is paired with high-sample, frequent (roughly monthly) surveys of individual respondents over a data collection period exceeding 3 years. These conditions likely contributed to a high degree of statistical power to detect relationships between impressions at the market-time level and recall measured at the individual level. Future evaluation research that aims to use impressions for evaluating multichannel campaigns should consider whether the available variation in both impressions and respondent data are sufficient to statistically capture relationships between impressions and respondent-level constructs.

Although our analysis was able to detect positive and statistically significant relationships between impressions and recall for each of the CTPP campaigns we measured, the pattern of predicted recall across observed impressions levels (Multimedia Appendix 1) suggests the overall magnitude of this relationship was modest. This may be due to a high level of background exposure to tobacco control media campaigns in California. Historically, California has a long track record of well-funded tobacco education media campaigns in addition to strong tobacco control policies [10,11]. These campaigns and policies have been implemented over a long period of time that precedes the time frame of our study. Given the likely high preexisting awareness of tobacco-related messages and related issues in California, the effects of ongoing awareness-raising media campaigns may be incremental. Therefore, our results should not be interpreted as a prescription of expected dose-responses for other campaigns that may occur in different contexts. Rather, the historical context of past campaign efforts and existing public awareness and knowledge of campaign-targeted messages should be considered when evaluating these types of campaigns.

### Limitations

There are several limitations to this analysis that should form the basis of next steps and recommendations for future research with similar data. First, impressions measure potential, not confirmed individual-level exposure. Although our results suggest that impressions are predictive of self-reported campaign recall, future research can further validate impressions as a proxy for exposure by applying them to evaluate campaign effects on other respondent-level outcomes, including campaign-targeted attitudes, beliefs, and behaviors.

Second, we did not examine the effects of channel-specific impressions on self-reported recall and therefore cannot make conclusions about the effects of different mixtures of media channels. This leaves unanswered questions about the equivalency of impressions across different media channels. For example, do impressions for video advertisements generate greater exposure rates than the same level of impressions for digital display advertisements? Furthermore, within video advertisements, does delivery through streaming services result in different exposure levels than those delivered by broadcast? Disentangling the unique contributions of each media channel is challenging, since creative elements including visuals, main messages, and calls to action, are usually very similar across all media channels used within a single campaign. The estimation of channel-specific effects would require the production of sufficiently different creative content across channels and more extensive coordination of ad buys set up for the purposes of evaluation tests. This level of coordination may not be possible for most campaigns, given limited resources and focus on concise sets of outcomes and objectives. Therefore, for purposes of addressing channel-specific effects, we recommend that future studies at least consider examining multiple campaigns that have differing creative content in addition to different mixtures of media channels.

An additional limitation is that although impressions have a straightforward definition, they are difficult to interpret, given the volumes that are delivered for individual campaigns, which often exceed tens of millions. To put results in greater context, future studies should consider reporting additional information, when available, about the campaign ad buys such as overall investment and expenditure levels for each media channel. These additional data can potentially facilitate helpful return on investment analysis as public health programs consider options for campaign ad buys with varying levels of purchased impressions.

Finally, we note that while the survey data are weighted to reflect the demographic profile of California, the data are not recruited using a statistical probability sample. Because the data are recruited from a large opt-in consumer panel, the unweighted data is skewed toward heavier representation of some groups such as older and more educated adults. This results in increased weighting for less represented groups and should be considered when interpreting the overall results.

### Conclusion

Measurement of digital media delivery and its application in the evaluation of multichannel public health campaigns is an emerging science. This study provides preliminary evidence that combined campaign impressions are predictive of overall campaign recall at the respondent level. Therefore, impressions may provide a valid alternative exposure variable that captures all media channels in evaluations of modern multichannel campaigns. Future research can build on this study by (1) exploring relationships between impressions and other campaign-targeted outcomes; (2) implementing study designs that attempt to distinguish the unique effects of each media channel; and (3) examining additional information on campaign investments to aid in the interpretation of evaluations that use impressions data.

## Appendix

Multimedia Appendix 1 Predicted values of campaign recall by observed all-campaign impressions levels among all adults in California aged 18-55 years, August 2019-December 2022.

## Abbreviations

CTPP: California Tobacco Prevention Program
DMA: designated market area
GRP: gross ratings point
LGBQ: lesbian, gay, bisexual, and queer/questioning
OR: odds ratio
TRP: target ratings point

## Data Availability

The dataset generated and analyzed during this study is not publicly available, but may be available from the sponsoring agency (CTPP) upon reasonable request.
