# Supplementary figures and images for: The Validity of Impressions as a Media Dose Metric in a Tobacco Public Education Campaign Evaluation: Observational Study
Source: J Med Internet Res. 2024 Nov 5;26:e55311. doi: 10.2196/55311 (PMC11576593; doi:10.2196/55311)

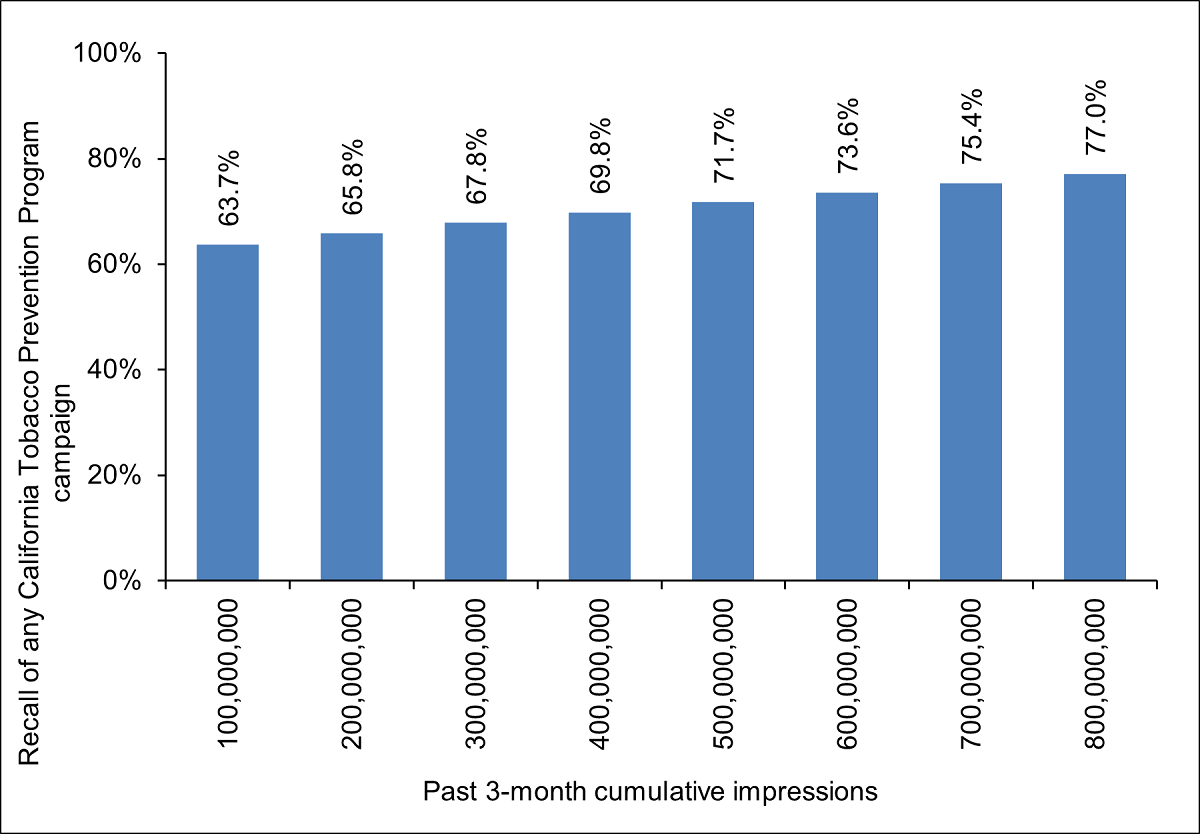

Supplement: Multimedia Appendix 1 [file jmir_v26i1e55311_app1.png]
